# Receptor-Type Guanylyl Cyclase at 76C (Gyc76C) Regulates *De Novo* Lumen Formation during *Drosophila* Tracheal Development

**DOI:** 10.1371/journal.pone.0161865

**Published:** 2016-09-19

**Authors:** Monn Monn Myat, Unisha Patel

**Affiliations:** 1 Department of Cell and Developmental Biology, Weill Cornell Medical College, New York, NY, United States of America; 2 Department of Biology, Medgar Evers College-City University of New York, Brooklyn, New York, United States of America; Texas A&M University, UNITED STATES

## Abstract

Lumen formation and maintenance are important for the development and function of essential organs such as the lung, kidney and vasculature. In the *Drosophila* embryonic trachea, lumena form *de novo* to connect the different tracheal branches into an interconnected network of tubes. Here, we identify a novel role for the receptor type guanylyl cyclase at 76C (Gyc76C) in *de novo* lumen formation in the *Drosophila* trachea. We show that in embryos mutant for *gyc76C* or its downsteam effector protein kinase G (PKG) 1, tracheal lumena are disconnected. Dorsal trunk (DT) cells of *gyc76C* mutant embryos migrate to contact each other and complete the initial steps of lumen formation, such as the accumulation of E-cadherin (E-cad) and formation of an actin track at the site of lumen formation. However, the actin track and E-cad contact site of *gyc76C* mutant embryos did not mature to become a new lumen and DT lumena did not fuse. We also observed failure of the luminal protein Vermiform to be secreted into the site of new lumen formation in *gyc76C* mutant trachea. These DT lumen formation defects were accompanied by altered localization of the Arf-like 3 GTPase (Arl3), a known regulator of vesicle-vesicle and vesicle-membrane fusion. In addition to the DT lumen defect, lumena of *gyc76C* mutant terminal cells were shorter compared to wild-type cells. These studies show that Gyc76C and downstream PKG-dependent signaling regulate *de novo* lumen formation in the tracheal DT and terminal cells, most likely by affecting Arl3-mediated luminal secretion.

## Introduction

**Fig 1:**
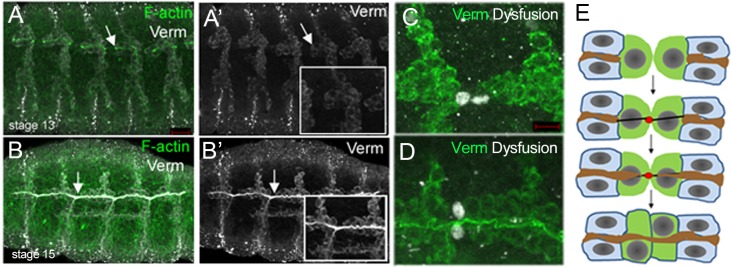
*De novo* lumen formation in the *Drosophila* trachea. In wild-type embryos dorsal trunk (DT) branches are initially blind-ended tubes with a central lumen outlined by F-actin (A and A’, arrows). New lumena form between pre-existing lumena to generate an interconnected network of tubes (B and B’, arrows). DT fusion cells (C and D, white) mediate fusion of the lumen labelled with Vermiform (Verm; green). Schematic diagram of the steps in DT lumen formation (E): fusion cells of opposing DT branches (green) accumulate E-cadherin (red) at the site of contact and form an actin and microtubule cytoskeletal track that spans the fusion cells. Lumena of the DT branches (brown) grow along the track towards the site of contact and the actin/microtubule track matures into a new lumen that connects the pre-existing lumena. Insets in A’ and B’ indicate magnified views of regions in A and B marked by arrows. Embryos in A and B were stained for F-actin (green) and Verm (white). Embryos in C and D were stained for Dysfusion (Dys; white) to label fusion cells and Verm (green) to label the lumen. Scale bars represent 5 μm.

Many of our essential organs, such as the lung, kidney and vasculature are tube-based structures where gases, nutrients and waste are transported through their respective lumena. For some tubular organs, lumena form *de novo* whereas for others, lumena form concomitantly with tube formation. Much of our understanding of *de novo* lumen formation has come from studies in the *Drosophila* embryonic trachea, a network of interconnected epithelial tubes that transport oxygen and other gases [1, 2]. As tracheal cells migrate out to form the primary branches, blunt-ended tubes with a sealed central lumen are initially formed (Fig 1A). A continuous tubular network is formed when specialized fusion cells at the tips of migrating branches contact each other’s partner in the adjacent segment and mediate *de novo* lumen formation and lumen fusion (Fig 1B–1D) [3, 4]. Lumen formation in the *Drosophila* trachea is a complex and highly regulated process involving precise coordination of cytoskeletal proteins, adhesion proteins and components of the vesicular trafficking machinery. During lumen formation fusion cells of opposing tracheal branches, such as the dorsal trunk (DT) contact each other through E-cadherin-mediated adhesion to form actin and microtubule tracks that prefigure the future luminal axis (Fig 1E). This is followed by growth of the pre-existing lumena along the track towards the new lumen site and subsequent expansion to form a lumen of a uniform size. Lumen formation requires targeted exocytosis and plasma membrane remodeling. These processes are mediated by the Arf-like 3 small GTPase (Arl3) which associates with microtubules and vesicles [5, 6], and the COPI coatomer complex that controls vesicular transport [7].

To identify genes required for tracheal development we previously performed a large scale chemical mutagenesis screen [8]. From this screen we identified a novel allele of the receptor type guanylyl cyclase at 76C (Gyc76C). Guanylyl cyclases (GCs) are a family of soluble and receptor-type enzymes that catalyze the conversion of GTP to cGMP in response to signals, such as nitric oxide (NO), peptide ligands and changes in intracellular calcium [9–12]. In *Drosophila*, Gyc76C regulates axon guidance by physically associating with the Semaphorin 1a receptor Plexin A [13, 14] whereas Gyc32E is involved in oogenesis and in egg chamber development [15]. Most of the effects of cGMP signaling are mediated by the activation of cGMP-dependent protein kinases (cGKs or PKGs) [9, 10, 16]. The two *Drosophila* cGMP-dependent kinases, PKG1/DG1 and PKG2/DG2 are encoded by the *pkd21D* and *foraging* (*for*) genes, respectively. *for* was recently shown to regulate the cytoplasmic-nuclear trafficking of the transcription factor Lola during *Drosophila* axon guidance [17]. In addition to a role in axon guidance, our previous studies showed that Gyc76C is required for salivary gland and muscle development in the *Drosophila* embryo. In *gyc76C* mutants, the salivary gland fails to migrate and the lumen is branched [18]. The accompanying defects in accumulation of the extracellular matrix (ECM) protein laminin and the integrin-adhesion receptor binding protein, talin suggest that the migration and lumen shape defects in *gyc76C* mutant glands may in part be due to defects in integrin-mediated adhesion to the ECM. We also showed that Gyc76C is required during muscle development for proper localization of integrins at sites of contact between the body wall muscles and tendon cells [19]. Consistent with our demonstration of a role for *gyc76C* and *for* in integrin-dependent adhesion, recent studies in the developing wing show that *gyc76C* and *for* regulate ECM-remodeling matrix metalloproteinases [20]. Although we reported *gyc76C* to be expressed in the developing trachea [19], it was previously not known what role *gyc76C* played in tracheal development. Here, we show that Gyc76C is required for *de novo* lumen formation in the dorsal trunk (DT) and terminal branches of the embryonic trachea, at least in part by controlling the intracellular localization of Arl3.

## Materials and Methods

### *Drosophila* Strains and Genetics

Canton-S flies were used as wild-type controls. *gyc76C*^*2388*^ was obtained by standard EMS mutagenesis as previously described [8]. *gyc76C*^*ex173*^ and UAS-*gyc76C*^*WT*^ lines were obtained from A. Kolodkin (Johns Hopkins University School of Medicine, Baltimore, MD). *UAS-gyc76CRNAi* and UAS-*pkg21D* RNAi lines were obtained from S. Davies (University of Glasgow, United Kingdom). *pkg21D*^*f05504*^ was obtained from the Exelixis collection at Harvard Medical School and is described in Flybase (http://flybase.bio.indiana.edu/). UAS-*mcd8GFP* was obtained from the Bloomington Stock Center and is described in FlyBase. *Arl3*^*CG6678*^ was obtained from L. Jiang (Oakland University, Rochester, MI). For tracheal-specific expression of the UAS constructs, we used the *breathless* (*btl*)-GAL4 driver.

### Immunocytochemistry

Embryo fixation and antibody staining were performed as previously described [21]. The following antisera were used at the indicated dilutions: rabbit Vermiform antiserum at 1:300 (a gift of S. Luschnig); rat Dysfusion antiserum at 1:200 (a gift of S. Crews); mouse 2A12 antiserum at 1:10 and rat E-cadherin antiserum at 1:20 (Developmental Studies Hybridoma Bank, DSHB; Iowa City, IA); guinea pig Arl3 antiserum at 1:200 (a gift of L. Jiang); mouse DSRF antiserum at 1:100 (Active Motif, Carlsbad, CA) and mouse β-galactosidase (β-gal) antiserum at 1:500 (Promega, Madison, WI). Appropriate biotinylated- (Jackson Immunoresearch Laboratories, Westgrove, PA), AlexaFluor 488-, 647- or Rhodamine- (Molecular Probes-Thermofisher Scientific, Waltham, MA) conjugated secondary antibodies were used at a dilution of 1:500. F-actin was detected with phalloidin (1:20; Invitrogen-Thermofisher Scientific) as previously described [22]. Stained embryos were mounted in Aqua Polymount (Polysciences, Inc., Warrington, PA) and thick (1 μm) fluorescence images were acquired on a Zeiss Axioplan microscope (Carl Zeiss) equipped with LSM 510 for laser scanning confocal microscopy at the Weill Cornell Medical College optical core facility (New York, NY).

### Quantification of terminal cell lumen length

Terminal cell lumen length was measured from the center of the DSRF-stained nucleus to the tip of the 2A12-stained lumen using Image J software (National Institute of Health, Bethesda, MD). A minimum of 10 lumena were measured for each genotype. Statistical analysis was done using Microsoft Excel.

## Results

**Fig 2:**
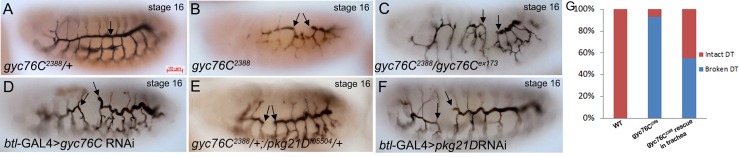
DT lumen defects in *gyc76C* and *pkg21D* mutant embryos. In *gyc76C*^*2388*^ heterozygous embryos (A) the DT lumen is continuous (A, arrow) whereas in homozygous siblings (B), *gyc76C*^*2388*^ *gyc76C*^*ex173*^ *trans*-heterozygous embryos (C), embryos expressing *gyc76C* RNAi in the trachea with *btl*-GAL4 (D), *gyc76C*^*2388*^ *pkg21D*^*f05504*^ *trans*-heterozygous embryos (E) and embryos expressing *pkg21D* RNAi in the trachea (F), the DT lumen is disconnected (B-F, arrows). Graph depicting percentage of intact (G, red) and broken (G, blue) DT lumena in wild-type embryos, *gyc76C*^*2388*^ homozygous embryos and *gyc76C*^*2388*^ homozygous embryos expressing wild-type *gyc76C* (*gyc76C*^*WT*^) in the trachea with *btl*-GAL4 (*gyc76C*^*WT*^ rescue in trachea). All embryos shown were stained for 2A12 to mark the tracheal lumen (dark brown) and β-galactosidase (β-gal) (brown) to distinguish heterozygous from homozygous embryos.

To determine *gyc76C* function in tracheal development we analyzed embryos mutant for *gyc76C*^*2388*^ [18] and *gyc76C*^*ex173*^[13]. In embryos heterozygous for a null allele of *gyc76C*, *gyc76C*^*2388*^, the DT lumen is a continuous structure (Fig 2A). By contrast, in *gyc76C*^*2388*^ homozygous embryos the lumen was disconnected at various points along the length of the DT (Fig 2B). Approximately 93% of *gyc76C*^*2388*^ homozygous embryos showed DT lumen defects compared to wild-type and heterozygous siblings that showed no defects (Fig 2G). Similarly, DT lumen defects were observed in embryos homozygous for *gyc76C*^*ex173*^, *trans*-heterozygous for *gyc76*^*ex173*^ and *gyc76C*^*2388*^ and embryos expressing *gyc76C* RNAi specifically in the trachea with the *breathless* (*btl*)-GAL4 driver (Fig 2C and 2D and data not shown). Similar to *gyc76C* mutant embryos, expression of RNAi to *pkg21D*, encoding the *Drosophila* cGMP-dependent protein kinase 1 (PKG1) resulted in a DT lumen defect (Fig 2F). Embryos *trans*-heterozygous for *gyc76C*^*2388*^ and a loss-of-function allele of PKG1, *pkg21D*^*f05504*^ also showed DT lumen defects, suggesting a strong genetic interaction between *gyc76C*^*2388*^ and *pkg21D* (Fig 2E).

**Fig 3:**
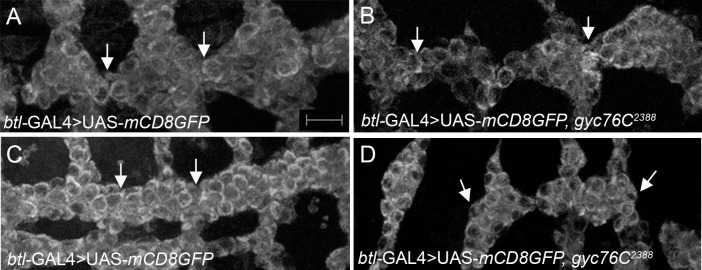
*gyc76C* mutant DT cells migrate but do not remain connected. DT cells of wild-type embryos expressing mCD8-GFP in the trachea with *btl*-GAL4 (A and C, arrows) migrate towards each other at stage 12 and remained connected by stage 15 (C). DT cells of *gyc76C*^*2388*^ homozygous embryos expressing mCD8GFP migrate towards each other at stage 12 (B, arrows) but do not remain connected to each other by stage 15 (D, arrows). All embryos shown were stained for GFP with those in B and D being also stained for β-gal (not shown). Scale bar in A represents 5 μm.

To test if the discontinuous tracheal lumen observed in *gyc76C* mutant embryos is due to a cell migration or a lumen fusion defect, we analyzed embryos expressing cytoplasmic mCD8-GFP specifically in the trachea of *gyc76C*^*2388*^ heterozygous and homozygous embryos. In *gyc76C*^*2388*^ heterozygous and homozygous embryos, DT cells of all embryos analyzed migrated normally to contact their counterparts in the neighboring tracheal metameres at stage 12 (Fig 3A and 3B). However, as embryogenesis progressed DT branches of *gyc76C*^*2388*^ homozygous embryos did not remain connected with many of them disconnected by stage 15, unlike their heterozygous siblings (Fig 3C and 3D). Thus, DT cells of *gyc76C*^*2388*^ mutant embryos migrated normally; however, because the lumena did not fuse, some branches remained separated and an interconnected tracheal network did not form.

We previously showed that *gyc76C* mRNA is expressed in the trachea from the onset of primary branch migration until the end of embryogenesis [19]. To test if *gyc76C* is required cell-autonomously in the trachea we expressed wild-type *gyc76C* (*gyc76C*^WT^) in the trachea of *gyc76C*^*2388*^ homozygous embryos with *btl*-GAL4. Expression of *gyc76C*^WT^ reduced the percentage of embryos with DT lumen fusion defects from 95% to 55% (Fig 2G). It is possible that lumen fusion defects persist because of an insufficient amount and/or temporal requirement of wild-type *gyc76C* expression. These data demonstrate that *gyc76C* acts in the trachea to regulate tracheal lumen formation.

### Actin and E-cadherin maturation defects in *gyc76C* and *pkg21D* mutant trachea

**Fig 4:**
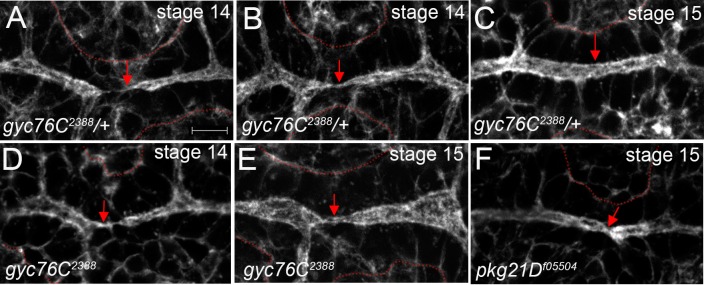
Actin track does not mature in *gyc76C* and *pkg21D* mutant trachea. In DT branches of *gyc76C*^*2388*^ heterozygous embryos (A-C), an actin track forms between fusion cells of opposing branches (A, arrow) followed by the maturation of the track (B, arrow) into a new lumen that is continuous with and of equal diameter as the pre-existing lumena (C, arrow). In *gyc76C*^*2388*^ homozygous embryos the actin track forms (D, arrow) but does not mature into a new lumen (E, arrow). In *pkg21D*^*f05504*^ mutant embryos the site of lumen fusion is constricted (F, arrow). All embryos were labeled for F-actin with phalloidin and β-gal (not shown). Dotted red line outlines the DT cells. Scale bar represents 5 μm.

During DT lumen formation an actin-rich track assembled between the fusion cells of two adjacent DT branches in *gyc76C*^*2388*^ heterozygous embryos as in wild-type embryos (Fig 4A and data not shown). This is followed by growth of the pre-existing lumena of opposing DT branches towards the site of contact between the fusion cells (Fig 4B). The actin track then matured into a new lumen that connected the pre-existing lumena (Fig 4C). In *gyc76C*^*2388*^ homozygous embryos, the actin track was assembled between the fusion cells in the same temporal manner as in heterozygous siblings (Fig 4D). However, the actin track of *gyc76C*^*2388*^ homozygous embryos did not mature into a new lumen at the stage when in heterozygous siblings the new lumen had already expanded to the same diameter as the pre-existing lumena (Fig 4E). We observed a similar defect in *pkg21D*^*f05504*^ mutant embryos where the lumen remained constricted at the site of fusion (Fig 4F).

**Fig 5:**
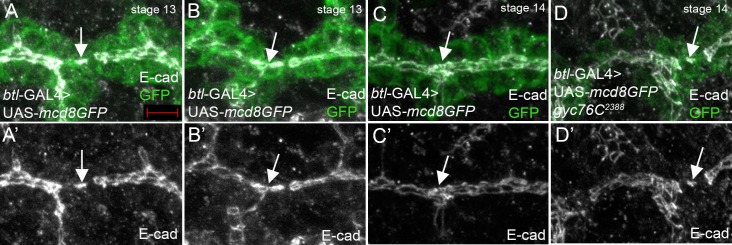
E-cad contact site failed to expand in *gyc76C* mutant embryos. In wild-type embryos expressing mcd8-GFP in the trachea with *btl*-GAL4 (A-C), E-cad initially accumulates as a patch at the site of contact between the two fusion cells (A and A’, arrows) and then becomes continuous with the pre-existing lumena (B, B’, C and C’, arrows). In *gyc76C*^*2388*^ homozygous embryos expressing tracheal mcd8-GFP (D) E-cad accumulates at the fusion cell contact site but does not expand to connect with the pre-existing lumena (D and D’, arrows). Embryos were stained for E-cad (white), GFP (green) and β-gal (not shown). Scale bar represents 5 μm.

In DT cells of wild-type embryos expressing mCD8-GFP in the trachea, the cell-cell adhesion protein, E-cadherin (E-cad) accumulated at the site of contact between the fusion cells at the onset of lumen formation (Fig 5A). E-cad then expanded to become continuous between the fusion cells and the adjacent DT cells (Fig 5B and 5C). *gyc76C*^*2388*^ mutant DT cells accumulated E-cad at the site of contact between the fusion cells; however, E-cad did not expand and remained at the initial contact site (Fig 5D). These data together demonstrate that Gyc76C is not required for the initial formation of the actin track or the accumulation of E-cad at the site of new lumen formation but is required for the maturation and expansion of the actin track and E-cad.

### Vermiform is not transported into the new lumen site in *gyc76C* mutant trachea

**Fig 6:**
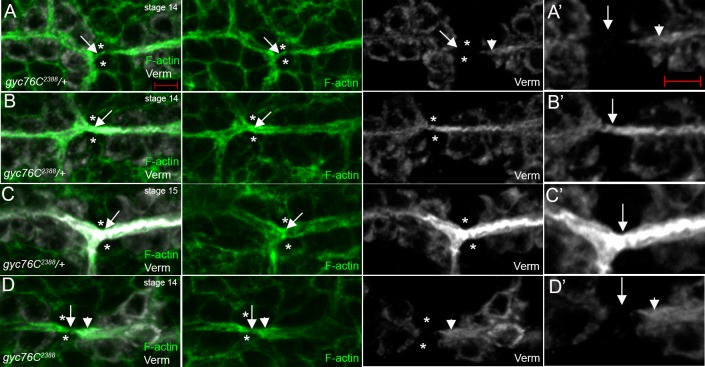
Verm is not transported into the lumen fusion site in *gyc76C* mutant trachea. In *gyc76C*^*2388*^ heterozygous embryos (A-C), Verm (A and A’, white) is initially absent from the site of new lumen formation (A and A’, arrow) marked by the actin track (A, green) that forms between the fusion cells (A, asterisks). As the new lumen forms (B and B’), and expands to the same diameter as the pre-existing lumena (C and C’, arrow), Verm is now present in the new lumen (B’ and C’, white). In *gyc76C*^*2388*^ homozygous embryos (D), Verm (D and D’, white) is absent from the site of lumen fusion (D and D’, arrows) although it is present at reduced levels in the pre-exisiting lumena (D and D’, arrowheads). Panels A’-D’ are magnified views of the fusion sites indicated by arrows in A-D, respectively. All embryos were stained for F-actin with phalloidin (green), Verm (white) and β-gal (not shown). Embryos in A, B and D are at stage 14 whereas the embryo in C is at stage 15. Scale bars represent 5 μm.

To test if *gyc76C* is required for membrane transport into the site of *de novo* lumen formation we analyzed the localization of Vermiform, a chitin-modifying enzyme [23]. In *gyc76C*^*2388*^ heterozygous embryos, as in wild-type embryos, Verm was initially found in the cytoplasm and pre-existing lumena of DT cells but not in the fusion cells (Fig 6A). As the actin track matured into a new lumen and the pre-existing lumena fused, Verm accumulated in the newly formed lumen but was absent from the cytoplasm of the fusion cells (Fig 6B). Even when the newly formed lumen expanded to the same diameter as the pre-existing lumena, Verm was not detected in the fusion cells (Fig 6C). These data show that luminal proteins such as Verm, that are not secreted by the fusion cells but instead by the neighboring DT cells are transported into the site of new lumen formation. In *gyc76C*^*2388*^ homozygous embryos, Verm was synthesized and secreted into the pre-existing lumena by the DT cells, albeit at a reduced level; however, Verm was not detected at the site where the new lumen should have formed (Fig 6D). Thus, *gyc76C* is required for delivery of the luminal protein Verm into the site of new lumen formation.

### *Gyc76C* genetically interacts with *Arl3* and controls Arl3 localization in DT fusion cells

**Fig 7:**
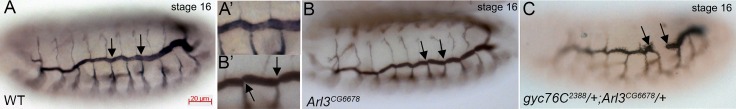
*Arl3* genetically interacts with *gyc76C* to control DT lumen fusion. In wild-type embryos (A) the DT lumen is continuous by stage 16 (A and A’, arrows). In embryos homozygous for *Arl3*^*CG6678*^ (B) the DT lumen is constricted at sites of fusion (B and B’, arrows) and in embryos *trans*-heterozygous for *gyc76C*^*2388*^ and *Arl3*^*CG6678*^ (C) DT lumena failed to fuse (C, arrow). All embryos shown are at stage 16 and were stained for 2A12 and β-gal (not shown). Panels A’ and B’ are magnified views of regions in A and B marked by arrows.

**Fig 8:**
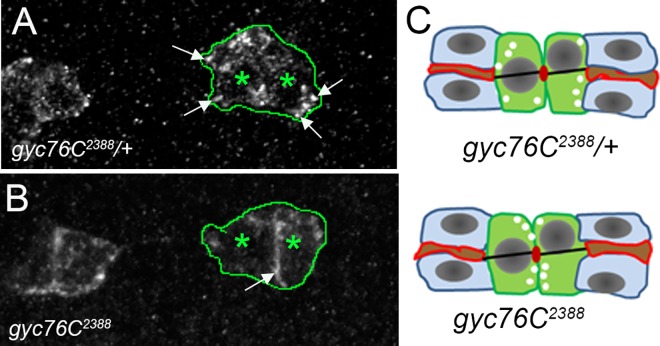
Arl3 is mislocalized in *gyc76C*^*2388*^ mutant fusion cells. In *gyc76C*^*2388*^ heterozygous embryos (A), Arl3 is found in a punctate pattern in the cytoplasm of the fusion cells (A, arrows). In *gyc76C*^*2388*^ homozygous embryos (B), Arl3 is predominantly found at contact sites between the two adjacent fusion cells (B, arrow). Diagram depicting mislocalization of Arl3-positive puncta in *gyc76C*^*2388*^ heterozygous embryos compared to homozygous siblings (C). Red outlines in C indicate E-cadherin-mediated cell-cell contact sites, the actin and microtubule track in black and fusion cells in green. Asterisks in A and B indicate fusion cells. Embryos in A and B were stained for Arl3 (white) and β-gal (not shown).

In embryos mutant for an Arf-like 3 GTPase (Arl3), DT lumena failed to fuse [5, 6]. This is thought to be due to a defect in vesicle-vesicle and vesicle-plasma membrane fusion. In embryos homozygous for a hypomorphic allele of *Arl3*, *Arl3*^*CG6678*^ [5] the DT lumen was continuous; however, the lumena were constricted at sites of fusion, unlike in wild-type embryos (Fig 7A and 7B). In embryos *trans*- heterozygous for *gyc76C*^*2388*^ and *Arl3*^*CG6678*^ DT lumena failed to fuse, like in *gyc76C*^*2388*^ mutant embryos (Fig 7C). In wild-type tracheal fusion cells endogenous Arl3 is found as cytoplasmic puncta and at the contact site between fusion cells [5, 6]. In *gyc76C*^*2388*^ heterozygous embryos, endogenous Arl3 was found as cytoplasmic puncta whereas in *gyc76C*^*2388*^ homozygous embryos, Arl3 was enriched at the contact sites between the fusion cells (Fig 8A–8C). Thus, loss of *gyc76C* altered the subcellular localization of Arl3 in DT fusion cells.

### Gyc76C is required for *de novo* lumen formation in terminal cells

**Fig 9:**
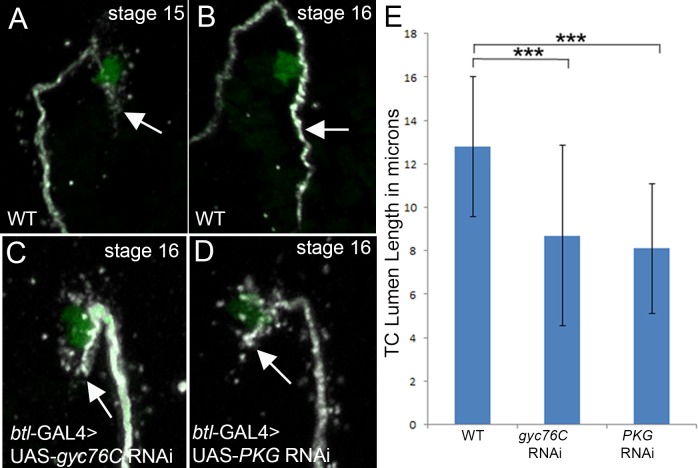
Gyc76C and PKG1 are required for terminal cell lumen elongation. In terminal cells of wild-type embryos (A and B) the TC lumen begins to form at stage 15 (A) and is elongated by stage 16 (B). In embryos expressing *gyc76C* RNAi (C) or *PKG1* RNAi (D) specifically in the trachea with *btl*-GAL4, TC lumena do not elongate (C and D, arrows). Quantification of TC lumen length shows that the lumena of trachea expressing *gyc76C* RNAi or *PKG1* RNAi are significantly shorter than those of wild-type trachea (E). Embryos in A-D were stained for DSRF (green) to label TC nuclei and 2A12 (white) to label the lumena. *** = p<0.01.

To test if *gyc76C* is required for *de novo* lumen formation in other tracheal branches we analyzed lumen formation in the terminal cells (TCs) which form a seamless intracellular lumen. Unlike the DT where new lumena form and connect with pre-existing lumena, TC lumen formation involves the inward growth of an intracellular lumen [24, 25]. We analyzed the dorsal terminal branches of wild-type embryos and embryos expressing RNAi to *gyc76C* or *pkdg21D* specifically in the trachea with the *btl*-GAL4 driver. In wild-type embryos the TC lumen elongated between stages 15 and 16 (Fig 9A and 9B). By contrast, in TCs where *gyc76C* or *pkg21D* have been knocked down with RNAi, the lumen was not elongated and an increased number of punctate structures were observed in the cytoplasm of the TCs (Fig 9C and 9D). Quantification of the TC lumen length showed significant reduction in *gyc76C* RNAi- and *pkdg21D* RNAi-expressing trachea compared to WT (Fig 9E). Thus, *gyc76C* and *pkg21D* are required for *de novo* lumen formation in the terminal branches.

## Discussion

We demonstrate in this study that Gyc76C and its downsteam effector PKG1 are required for *de novo* lumen formation in the *Drosophila* embryonic trachea. During DT lumen fusion, the actin track and the initial site of E-cad-mediated contact between fusion cells formed normally; however, a new lumen did not form and the pre-existing lumena of adjacent DT branches did not fuse. The presence of the secreted protein Verm in the DT cells and lumena but not at the lumen fusion site suggests that the *gyc76C* lumen defect is due at least in part to defects in the transport and/or fusion of vesicles at the lumen fusion site. Gyc76C may control vesicle transport and/or fusion through Arl3 GTPase which is known to be required for vesicle-vesicle and vesicle-plasma membrane fusion [5, 6]. The altered localization of Arl3 in *gyc76C* mutant fusion cells may prevent proper routing, delivery or fusion of vesicles necessary for the formation of a new lumen. Arl3 is known to regulate the localization of the exocyst subunit Sec5 at contact points between fusion cells and along the actin track, and has been shown to interact with microtubules [5, 6]. Thus, Gyc76C-dependent regulation of Arl3 localization in tracheal fusion cells maybe important for exocyst-mediated vesicle transport along actin and/or microtubule tracks and membrane fusion.

Our finding that *gyc76C* and *pkg21D* regulate *de novo* lumen formation in both the DT and TCs suggests a common mechanism for *de novo* lumen formation in different branches of the *Drosophila* trachea. Despite the distinct morphology of the DT lumen and the TC lumen, there are conserved features of lumen formation between the two tubular structures. For example, similar to the actin and microtubule tracks that form during DT lumen fusion, actin and microtubules are organized along the elongating lumen of the TC [25, 26]. Moreover, the exocyst complex is required for membrane trafficking events during lumen formation in both the DT and the TC [5, 6, 27]. Thus, *gyc76C* and *pkg21D* likely regulate *de novo* lumen formation in both the DT and TCs through a common mechanism.

We previously reported that *gyc76C* is required for proper localization of the βPS integrin subunit at the myotendinous junctions of developing somatic muscle, and for laminin localization around the migrating salivary gland [18, 19]. Since newly synthesized integrin and laminin proteins are transported through the endomembranous system, our data on Gyc76C function in the developing trachea, somatic muscle and salivary gland indicate a conserved role in vesicle transport and/or fusion. This is consistent with studies in mammalian cells where cGMP dependent protein kinases are shown to regulate a number of different membrane trafficking events, such as phagocytosis [28] and synaptic vesicle trafficking [29–31]. Moreover, loss of cGKII, the mammalian homolog of PKG2, results in intestinal secretory defects (Pfeifer et al., 1996). Interestingly, Arf and Arf-like GTPases, like guanylyl cyclases and cGMP dependent protein kinases, are requried for exocytosis in neuronal cells [32, 33]. Thus, cGMP signaling through Arl GTPases may be a conserved mechanism for regulating membrane transport in a number of distinct cell types.
